# The use of lateral wedge insoles delays osteoarthritis progression and improves clinical outcomes in medial meniscus posterior root repair

**DOI:** 10.1002/jeo2.70141

**Published:** 2025-01-20

**Authors:** Koki Kawada, Yusuke Yokoyama, Yuki Okazaki, Masanori Tamura, Toshifumi Ozaki, Takayuki Furumatsu

**Affiliations:** ^1^ Department of Orthopaedic Surgery Okayama University Graduate School of Medicine, Dentistry, and Pharmaceutical Sciences Okayama Japan; ^2^ Department of Orthopaedic Surgery Japanese Red Cross Okayama Hospital Okayama Japan

**Keywords:** healing status, lateral wedge insole, meniscus extrusion, osteoarthritis, posterior root tear

## Abstract

**Purpose:**

The purpose of this retrospective study was to evaluate the efficacy of using a lateral wedge insole (LWI) during the first 3 months after medial meniscus posterior root (MMPR) repair.

**Methods:**

Overall, 179 patients were categorized into LWI use (LWI group, 90 patients) and nonuse (control group, 89 patients) groups. Patients in the LWI group were instructed to wear an LWI from the initiation of load bearing up to 3 months postoperatively. Medial meniscus extrusion (MME) was evaluated preoperatively and 1 year postoperatively, Kellgren–Lawrence (KL) grade and clinical scores were evaluated preoperatively and 2 years postoperatively, and second‐look arthroscopic meniscal healing scores were evaluated at 1 year postoperatively.

**Results:**

The proportion of patients with KL grade progression at 2 years postoperatively was significantly lower in the LWI group than in the control group (23.3% vs. 39.3%; *p* = 0.024). Change in the MME at 1 year postoperatively was significantly smaller in the LWI group than in the control group (1.1 ± 1.2 vs. 1.6 ± 1.4 mm; *p* = 0.042). The Lysholm score (*p* = 0.003) and Knee Injury and Osteoarthritis Outcome Scores‐sport and recreation function (*p* = 0.027) at 2 years postoperatively were significantly superior in the LWI group than in the control group. The arthroscopic meniscal healing score after 1 year was not significantly different between the LWI and control groups (total score, 7.6 ± 1.1 vs. 7.4 ± 1.3 points; *p* = 0.732). The anteroposterior width of the repaired posterior root at 1 year second‐look evaluation was significantly broader in the LWI group than in the control group (7.7 ± 1.6 vs. 6.9 ± 1.6 mm; *p *= 0.001).

**Conclusions:**

The use of LWI is an effective way to delay postoperative osteoarthritis progression and improve clinical outcomes after MMPR repair.

**Level of Evidence:**

Level III.

**Abbreviations**
AP: anteroposterior
BMI: body mass index
HTO: high tibial osteotomy
ICC: intraclass correlation coefficients
IKDC: International Knee Documentation Committee
KL: Kellgren–Lawrence
KOOS: Knee Injury and Osteoarthritis Outcome Score
LWI: lateral wedge insole
MME: medial meniscus extrusion
MMPR: medial meniscus posterior root
MMPRTs: medial meniscus posterior root tears
MRI: magnetic resonance imaging
OA: osteoarthritis
TCS: two cinch stitches
ΔMME: changes in the medial meniscus extrusion

## INTRODUCTION

Medial meniscus posterior root (MMPR) tears (MMPRTs) often develop from minor trauma in middle‐aged and older adults, and most cases are associated with a sudden, painful popping of the posteromedial knee joint [11]. Recently, conservative treatment for MMPRTs has been reported to have poor long‐term outcomes, reemphasizing the importance of repair for MMPRTs [20, 23]. Pullout repair for MMPRTs has shown favourable clinical outcomes in the mid‐ to long‐term [1, 10]. However, even pullout repair for MMPRTs has not completely prevented the progression of knee osteoarthritis (OA) [1, 13]. More recently, high tibial osteotomy (HTO) has increasingly been performed for MMPRTs with varus alignment of the lower extremities [24, 31]. HTO is a remarkably effective [21] but highly invasive procedure compared to pullout repair and requires more attention to complications such as fractures, nonunion and infection [7, 25, 26].

Lateral wedge insoles (LWI) are noninvasive and relatively inexpensive and often used for conservative treatment of knee OA. LWI use has been reported to reduce knee adduction moment, lateral thrust and medial meniscus extrusion (MME) [9]. Additionally, it has been reported that the use of LWI for 3 months in early knee OA reduced the progression of MME [8]. However, research on the use of LWI after meniscal surgery is limited, and its use after meniscectomy has been reported to be ineffective [2]. To date, the use of LWI after pullout repair for MMPRTs has not been reported. This approach, unlike meniscectomy, is expected to promote successful recovery of the repaired posterior roots.

This study aimed to evaluate the effect of LWI use during the first 3 months postoperatively on the postoperative outcomes of pullout repair for MMPRTs. We hypothesized that the use of LWI would result in a superior recovery of the repaired posterior roots, delay postoperative OA progression and improve postoperative clinical scores.

## MATERIALS AND METHODS

### Patients

This retrospective study was approved by the Ethics Committee of Okayama University (No. 1857) and was conducted in accordance with the Declaration of Helsinki. Written informed consent was obtained from all patients.

As the standard at our facility, a complete MMPRT (MMPRT classification type 2–5 [22]) is indicated for pullout repair as soon as possible. For a partial MMPRT (MMPRT classification type 1), conservative treatment is performed first, and pullout repair is indicated for patients with persistent pain. However, a femorotibial angle > 180°, a Kellgren–Lawrence (KL) grade ≥ 3 and massive cartilage lesions (modified Outerbridge grade ≥ III) are contraindications for pullout repair and HTO, unicompartmental knee arthroplasty and total knee arthroplasty have been suggested as treatments. No contraindications for pullout repair were observed based on age, weight, body mass index (BMI) or patient's activity level.

**Figure 1:**
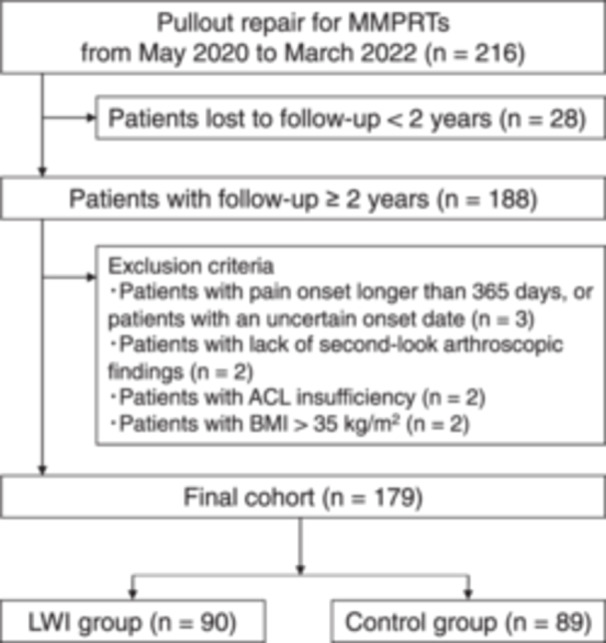
Flowchart of the study protocol. ACL, anterior cruciate ligament; BMI, body mass index; LWI, lateral wedge insole; MRI, magnetic resonance imaging; MMPRT, medial meniscus posterior root tear.

Between May 2020 and March 2022, 216 patients underwent pullout repair for MMPRTs (Figure 1). Of these, we collected data from 188 patients, excluding those lost to follow‐up <2 years (*n* = 28). Furthermore, patients with chronic MMPRT of unknown onset or >1 year from onset (*n* = 3), lack of second‐look arthroscopic findings (*n* = 2), anterior cruciate ligament insufficiency (*n* = 2) and a BMI > 35 kg/m^2^ (*n* = 2) were excluded for standardization of data and to eliminate intergroup differences. Finally, a total of 179 patients were classified into use of LWI (LWI group, 90 patients) and nonuse of LWI (control group, 89 patients) groups.

### LWI

**Figure 2:**
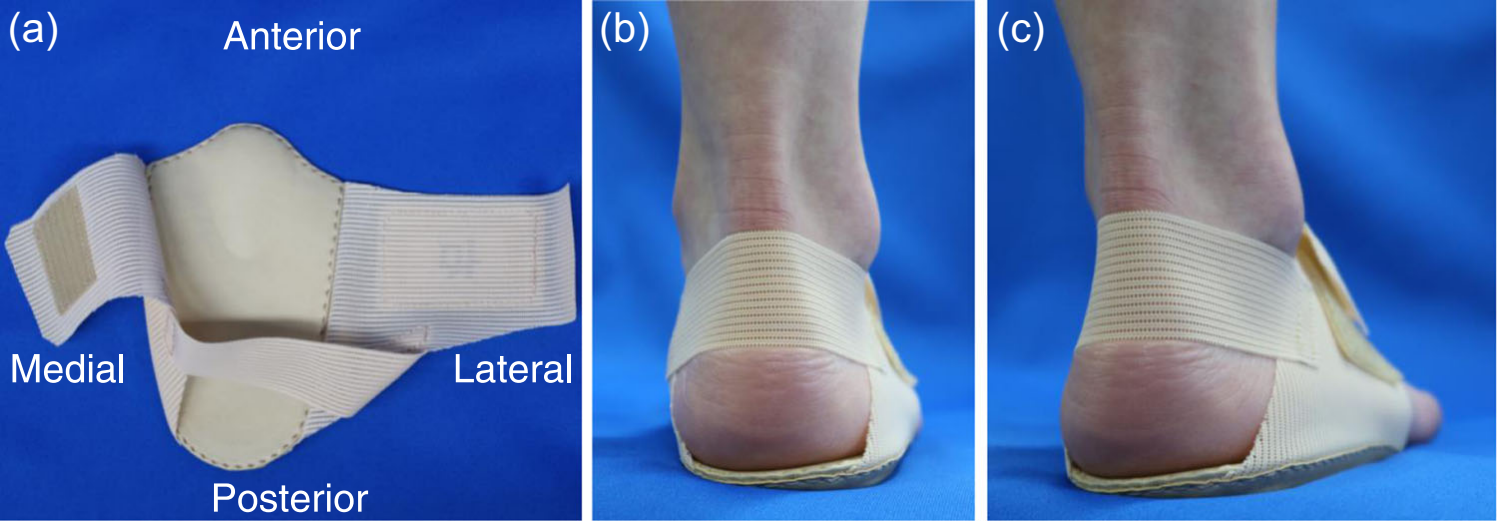
The LWI (right foot). An LWI with an 8‐mm height was created for each patient. It is shaped to cover only the rear foot area and is designed to be secured with a band. (a) Top, (b) rear and (c) posterolateral view of LWI. LWI, lateral wedge insole.

After April 2021, we recommended that the use of LWI be made for all patients after surgery, and only used LWI for patients who gave consent. A prosthetist created an LWI based on each patient's footprint (Figure 2). The LWI was designed with an 8‐mm lateral height and an elevation of approximately 5°–6° laterally, depending on the patient's plantar width. In addition, it was shaped to cover only the rear foot area and was designed to be secured with a band. Patients were instructed to use the LWI at all times when applying weight, both indoors and outdoors. During weekly rehabilitation, all patients received instructions and checks on the use of the LWI, and they were required to wear the LWI securely for 12 weeks after surgery.

### Surgical technique and rehabilitation protocol

All surgeries were performed by a single well‐experienced orthopaedic surgeon. For all patients, an outside‐in pie‐crusting technique was used to enlarge the medial knee compartment [5, 14]. The surgical technique was a pullout repair using two simple stitches or cinch stitches (TCS) alone or a combination of TCS and the posterior anchoring technique [32]. The stitches were performed with two No. 2 Ultrabraid sutures (Smith & Nephew), penetrating the MMPR. A 4.0 mm tibial foramen was created, and the sutures were pulled out. Pullout sutures were fixed with a bioabsorbable screw (Biosure RG, Smith & Nephew) and secured with a 5.0 mm cannulated screw as an anchor screw. In the posterior anchoring technique, which was additionally performed from December 2020 to July 2021, a bone tunnel was drilled posteriorly into the tibia, and fixation was added to the bone tunnel and the bottom of the posterior meniscal horn using the JuggerStitch™ meniscal repair device (Zimmer Biomet).

The affected knee was immobilized and unloaded during the first postoperative week. From 1 week postoperatively, weight‐bearing was increased by 20 kg, and the knee flexion angle was increased by 30° each week. Until 12 weeks postoperatively, the knee flexion was limited to 120°. Thereafter, no restrictions were imposed. Patients in the LWI group were instructed to wear the LWI only on the affected side from 1 up to 12 weeks postoperatively.

### Radiography and magnetic resonance imaging (MRI) assessment

**Figure 3:**
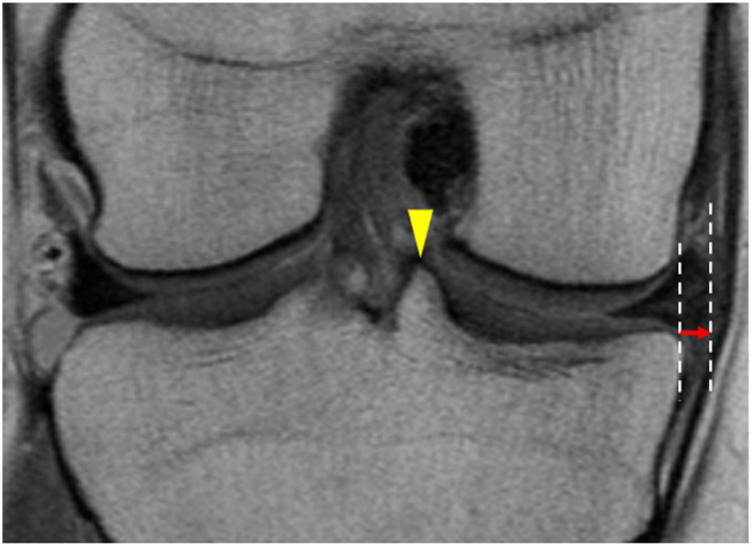
MME measurement using the bony landmark method (right knee). The MME is determined by measuring the horizontal distance (red arrow) between the medial point of the tibial plateau and the medial point of the medial meniscus in the slice where the highest medial tibial eminence (yellow arrowhead) is visible. MME, medial meniscus extrusion.

Radiography was performed preoperatively and 2 years postoperatively. The KL grade was evaluated using a Rosenberg view radiograph. MRI was performed preoperatively and 1 year postoperatively. MME was defined as the distance from the medial margin of the tibia, excluding the osteophytes, to the medial margin of the medial meniscus (Figure 3). The MME was measured using coronal MRI slices with the highest medial tibial eminence. Change in the MME (ΔMME) was calculated as the MME 1 year postoperatively minus the MME preoperatively. The KL grade and MME were measured twice by two independent observers (K. K. and M. T.). Intraclass correlation coefficients (ICC) were used to examine the intra‐ and interobserver reliabilities of the measurements. The ICC for KL grade and MME measurements were 0.861 and 0.925 for intraobserver repeatability and 0.793 and 0.937 for interobserver repeatability, respectively.

### Clinical scores

The following clinical scores were obtained preoperatively and 2 years postoperatively: Lysholm score, Tegner activity score, Knee Injury and Osteoarthritis Outcome Scores (KOOS), International Knee Documentation Committee (IKDC) score and pain visual analogue scale score.

### Second‐look arthroscopy

Second‐look arthroscopy was performed at 1 year postoperatively. The healing status of the repaired posterior roots was assessed using the (1) anteroposterior (AP) width score (0–4 points), (2) stability score (0–4 points) and (3) synovial coverage score (0–2 points) [4]. The total scores ranged from 0 to 10. Additionally, the absolute AP width of the repaired posterior roots was evaluated in millimetres (mm). This evaluation was performed by a single well‐experienced orthopaedic surgeon who performed the initial surgery. We performed second‐look arthroscopy in all patients who gave their consent, at the same time as the removal of the anchor screws.

### Statistical analysis

Statistical analyses were performed using the EZR software (Saitama Medical Centre). Normality of distribution was assessed using the Shapiro–Wilk test, which revealed a parametric distribution for MME and a nonparametric distribution for patient characteristics, KL grade, ΔMME, clinical scores and arthroscopic meniscal healing score.

The comparison of the LWI and control groups in terms of sex, surgical technique and MMPRT classification was conducted using Fisher's exact test. The comparison of the LWI and control groups in terms of age, height, body weight, BMI, duration from injury to operation, preoperative femorotibial angle, KL grade, ΔMME, clinical scores and arthroscopic meniscal healing score was conducted using the Mann–Whitney U test. The comparison of MME between the LWI and control groups was performed using an unpaired *t* test. In addition, the comparison of clinical scores preoperatively and 2 years postoperatively in each group was performed using the Wilcoxon signed‐rank test. Statistical significance was set at *p* < 0.05.

## RESULTS

The patient characteristics were not significantly different between the two groups (Table 1). The preoperative femorotibial angle did not differ significantly between the two groups (*p* = 0.994; Table 1). In addition, the MMPRT classification by LaPrade et al. [22] did not differ significantly between the two groups (*p* = 0.473; Table 1).

**Table 1:** Patient characteristics and surgical technique.

	LWI group (*n* = 90)	Control group (*n* = 89)	*p* Value
Sex, male/female	14/76	18/71	n. s.
Age, years	64.6 ± 8.3	66.4 ± 8.3	n. s.
Height, m	1.56 ± 0.07	1.56 ± 0.07	n. s.
Body weight, kg	62.4 ± 10.7	62.8 ± 12.3	n. s.
Body mass index, kg/m^2^	25.5 ± 3.6	25.8 ± 3.8	n. s.
Duration from injury to operation, days	69 ± 57	63 ± 55	n. s.
Preoperative femorotibial angle, °	178 ± 1	178 ± 2	n. s.
MMPRT classification, 1/2/3/4/5	9/73/0/8/0	11/74/0/4/0	n. s.
Surgical technique, TS/TS with PA technique	69/21	64/25	n. s.

*Note*: Data are displayed as mean ± standard deviation or number.

Abbreviations: LWI, lateral wedge insoles; MMPRT, medial meniscus posterior root tear; PA, posterior anchoring; TS, two stitches.

KL grade preoperatively and at 2 years postoperatively did not differ between the two groups, but the proportion of patients with KL grade progression was significantly lower in the LWI group than in the control group (*p* = 0.024; Table 2). MME preoperatively and at 1 year postoperatively did not differ between the two groups; however, ΔMME was significantly smaller in the LWI group (*p* = 0.042; Table 2).

**Table 2:** KL grade and MME comparison between the LWI and control groups.

	LWI group (*n* = 90)	Control group (*n* = 89)	*p* Value
Preoperative KL grade, 0/1/2/3/4	0/35/55/0/0	0/40/49/0/0	n. s.
2 years postoperative KL grade, 0/1/2/3/4	0/18/68/4/0	0/10/74/5/0	n. s.
KL grade progression, *n* (%)	21 (23.3)	35 (39.3)	**0.024** *
Preoperative MME, mm	4.3 ± 1.0	4.2 ± 1.2	n. s.
1‐year postoperative MME, mm	5.4 ± 1.5	5.8 ± 1.7	n. s.
ΔMME, mm	1.1 ± 1.2	1.6 ± 1.4	**0.042** *

*Note*: Data are displayed as mean ± standard deviation.

Abbreviations: KL, Kellgren–Lawrence; LWI, lateral wedge insole; MME, medial meniscus extrusion; ΔMME, change in the medial meniscus extrusion.

* Statistically significant.

Preoperative clinical scores did not significantly differ between the two groups, but clinical scores at 2 years postoperatively were significantly superior in the LWI group with regard to the Lysholm score (*p* = 0.003) and KOOS‐sport and recreation function (*p* = 0.027; Table 3).

**Table 3:** Pre‐ and postoperative clinical scores comparison between the LWI and control groups.

	LWI group	Control group	*p* Value
Lysholm score			
Preoperative	60.9 ± 12.2	59.9 ± 12.8	n. s.
Postoperative	89.4 ± 4.7	87.7 ± 4.9	**0.003** *
*p* Value	**<0.001** *	**<0.001** *	
Tegner activity score			
Preoperative	1.8 ± 0.8	1.8 ± 0.8	n. s.
Postoperative	3.0 ± 0.4	3.1 ± 0.5	n. s.
*p* Value	**<0.001** *	**<0.001** *	
KOOS‐pain			
Preoperative	61.2 ± 15.2	59.8 ± 16.6	n. s.
Postoperative	88.4 ± 8.9	88.2 ± 9.6	n. s.
*p* Value	**<0.001** *	**<0.001** *	
KOOS‐symptoms			
Preoperative	64.6 ± 16.8	63.0 ± 20.1	n. s.
Postoperative	84.6 ± 8.7	85.2 ± 11.0	n. s.
*p* Value	**<0.001** *	**<0.001** *	
KOOS‐ADL			
Preoperative	70.8 ± 14.1	68.2 ± 17.0	n. s.
Postoperative	90.1 ± 7.7	89.1 ± 9.0	n. s.
*p* Value	**<0.001** *	**<0.001** *	
KOOS‐Sport/Rec			
Preoperative	26.4 ± 20.0	26.1 ± 23.9	n. s.
Postoperative	61.0 ± 24.7	53.7 ± 27.3	**0.027** *
*p* Value	**<0.001** *	**<0.001** *	
KOOS‐QOL			
Preoperative	34.1 ± 18.9	34.6 ± 21.2	n. s.
Postoperative	64.6 ± 17.1	63.3 ± 18.7	n. s.
*p* Value	**<0.001** *	**<0.001** *	
IKDC score			
Preoperative	40.2 ± 14.0	38.4 ± 16.4	n. s.
Postoperative	69.1 ± 11.8	68.8 ± 13.0	n. s.
*p* Value	**<0.001** *	**<0.001** *	
Pain score (VAS)			
Preoperative	42.1 ± 25.2	44.3 ± 24.7	n. s.
Postoperative	9.7 ± 11.1	11.7 ± 13.5	n. s.
*p* Value	**<0.001** *	**<0.001** *	

*Note*: Data are displayed as mean ± standard deviation.

Abbreviations: ADL, activities of daily living; IKDC, International Knee Documentation Committee; KOOS, Knee Injury and Osteoarthritis Outcome Score; LWI, lateral wedge insole; QOL, quality of life; Sport/Rec, Sport and recreation function; VAS, visual analogue scale.

* Statistically significant.

The arthroscopic meniscal healing score did not differ significantly between the two groups. However, the absolute AP width of the repaired posterior roots was significantly broader in the LWI group (*p* = 0.001; Table 4).

**Table 4:** Arthroscopic meniscal healing score comparison between the LWI and control groups.

	LWI group	Control group	*p* Value
Total score, points	7.6 ± 1.1	7.4 ± 1.3	n. s.
AP width score, points	4.0 ± 0.3	3.9 ± 0.5	n. s.
Stability score, points	2.5 ± 0.8	2.6 ± 0.8	n. s.
Synovial coverage score, points	1.1 ± 0.8	0.9 ± 0.5	n. s.
Absolute AP width of the repaired posterior root, mm	7.7 ± 1.6	6.9 ± 1.6	**0.001** *

*Note*: Data are displayed as mean ± standard deviation.

Abbreviations: AP, anteroposterior; LWI, lateral wedge insole.

* Statistically significant.

## DISCUSSION

The key finding of this study was that using LWI during the first 3 months postoperatively of pullout repair for MMPRTs was associated with a broader AP width of the repaired posterior root, reduced the progression of MME at 1 year postoperatively and slowed the KL grade progression at 2 years postoperatively, resulting in a better clinical scores.

The rehabilitation after repair for MMPRTs has not yet been established. In a recent systematic review, eight of the 12 papers reported partial weight bearing within 1–4 weeks, and four papers reported 6 weeks of nonweight bearing [19]. While partial weight‐bearing and range‐of‐motion restrictions for more than 6 weeks are more likely to achieve better MMPR healing, they are also thought to be more likely to cause problems in daily life and muscle weakness. It has been reported that improvements in quadriceps muscle strength after MMPR repair are important for clinical outcomes [12]. Recent biomechanical studies have reported that partial weight‐bearing of up to 50% of body weight places little additional stress on the MMPR or medial femoral tibial joint [29]. In our study, partial weight bearing was started 1 week after surgery, and the weight was gradually increased thereafter, with full weight bearing achieved by 4 weeks after surgery, a relatively early rehabilitation protocol compared with previous reports.

LWI is used for conservative treatment of early‐onset knee OA. The mechanism of the effect produced by the LWI is the verticalization of the loading axis from the hip joint to the calcaneus and not to correct a varus deformity, such as the restoration of the femorotibial angle [33]. However, verticalization of the load axis has been reported to decrease the lateral thrust and knee adduction moment [28]. Stress reduction of the medial compartment using an LWI is expected to positively affect the recovery of the repaired posterior roots. In this study, these mechanisms may have inhibited the progression of MME, maintained the AP width of the repaired posterior roots and resulted in more favourable clinical scores.

MME is associated with knee OA progression [3]. Furthermore, it has been reported that there is a significant correlation between MME progression and medial joint space narrowing after MMPR repair [15]. Therefore, one goal of MMPR repair is to prevent the progression of MME. However, pullout repair for MMPRTs has not completely prevented the progression of MME in certain cases [16]. In this study, the use of LWI for the first 3 months postoperatively resulted in less progression of MME than in the group that did nonuse of LWI. The fact that the use of LWI was able to delay the MME progression, although not completely, is thought to be the reason for the delay in the progression of the medial joint space narrowing and the reduction in the KL grade progression.

No consensus exists on the appropriate LWI angle; nevertheless, 4°–6° has been reported as appropriate for the effectiveness and comfort of use [30]. We commissioned a prosthetist to create a custom‐made LWI for each patient, which was designed to be approximately 5°–6° higher laterally. Discussions regarding the appropriate duration of LWI use are limited. Kawai et al. studied the healing process of lesions in the vascular portion of the meniscus in dogs. After 12 weeks, the maximum strength of the repaired tissue reached 80% that of the control meniscus [18]. Additionally, it has been reported that the use of LWI for 3 months in early knee OA reduced the progression of MME [8]. Considering this, along with our 3 months rehabilitation protocol, patients were instructed to wear the LWI for 3 months in this study. The LWI used in this study was designed and shaped to cover only the rear foot area and could be secured with a band, aiming to reduce stress on the posteromedial compartment of the knee joint. Patients were permitted to wear shoes while using the LWI, both indoors and outdoors. All patients showed good compliance with the LWI design. The angle and duration of LWI use may also influence the progression of MME and warrant further examination.

Regarding the clinical scores in this study, the Lysholm score and KOOS‐sport and recreation function were significantly superior in the LWI group. Nie et al. reported that Lysholm and IKDC scores were significantly better in a group that was able to suppress the progression of MME [27]. In addition, Kawada et al. reported that there was a significant correlation between the progression of MME and clinical scores 3 years after surgery [17]. In this study, it is possible that the use of LWI delayed the progression of MME, and this may have led to a slight improvement in clinical scores. However, the difference between the two groups was only 1.7 points for the Lysholm score and only 7.3 points for the KOOS‐sport and recreation function. Therefore, it is highly likely that the difference in these clinical scores will not be noticed in clinical settings. Additionally, in this study, we evaluated nine clinical scores, but the LWI group was superior in only two of them. Thus, it may be important to note that the clinical scores after pullout repair for MMPRTs were good regardless of whether or not LWI was used.

The healing status of the repaired posterior roots in second‐look arthroscopy has been reported to predict mid‐term MME and clinical scores [17]. Additionally, a smaller progression of MME has been reported in patients with a broader absolute AP width of the repaired posterior roots [6]. In this study, both the LWI and control groups scored well, with similar total, width, stability and synovial coverage scores between the two groups. However, the absolute AP width of the repaired posterior roots was significantly broader in the LWI group. The broader the AP width of the repaired posterior roots, the more advantageous it is expected to be in terms of shock absorption, load distribution and stability of the knee joint, which are important roles of the meniscus. Accordingly, in this study, MME progression may have been reduced and clinical scores may have been further improved.

This study has some limitations. First, it was a retrospective study. Second, it was a short‐term evaluation; the MRI was performed at 1 year postoperatively, and the clinical scores were evaluated 2 years postoperatively. Further long‐term follow‐up results will be required. Third, it included patients who underwent an additional procedure of a posterior anchoring technique, mainly performed from December 2020 to April 2021, albeit with no significant difference in the number of patients undergoing this additional procedure between the two groups. Fourth, we excluded two cases with a BMI > 35 kg/m^2^ that were within the indications for pullout repair to adjust for the patient characteristics between the two groups. This may have been a selection bias that may have affected the results. Fifth, the second‐look arthroscopy could not be evaluated blindly with or without the use of LWI. Sixth, the KL grade is a subjective evaluation. The ICC for the KL grade in this study was >0.70, but this was lower than the ICC for MME, which was >0.90.

## CONCLUSIONS

The use of LWI during the first 3 months after MMPR repair was associated with a broader AP width of the repaired posterior roots and reduced the progression of MME at 1 year postoperatively. Additionally, the clinical scores were superior, and the KL grade progression was lower at 2 years postoperatively. The use of LWI is an effective way to delay postoperative OA progression and improve clinical outcomes after MMPR repair.

## AUTHOR CONTRIBUTIONS

Takayuki Furumatsu and Koki Kawada conceptualized this study and performed the documentation. All authors performed data collection and analysis. All authors commented on the first draft of the manuscript and approved the final draft.

## CONFLICT OF INTEREST STATEMENT

The authors declare no conflicts of interest.

## ETHICS STATEMENT

This study was conducted in accordance with the principles of the Declaration of Helsinki. The study was approved by the Ethics Committee of the Okayama University (No. 1857). Written informed consent was obtained from all patients.

## Data Availability

The data that support the findings of this study are available from the corresponding author, upon reasonable request.
